# Diagnostic and prognostic value of Bcl-2 in uterine leiomyosarcoma

**DOI:** 10.1007/s00404-022-06531-2

**Published:** 2022-03-28

**Authors:** Antonio Travaglino, Antonio Raffone, Diego Raimondo, Annarita Gencarelli, Italia Esposito, Cinzia Gallo, Francesco Paolo Improda, Salvatore Giovanni Vitale, Antonio Mollo, Paolo Casadio, Renato Seracchioli, Fulvio Zullo, Luigi Insabato

**Affiliations:** 1 Anatomic Pathology Unit, Department of Advanced Biomedical Sciences, School of Medicine, University of Naples Federico II, Naples, Italy; 2 Division of Gynaecology and Human Reproduction Physiopathology, Department of Medical and Surgical Sciences (DIMEC), IRCCS Azienda Ospedaliero-Univeristaria Di Bologna. S. Orsola Hospital, University of Bologna, Via Massarenti 13, 40138 Bologna, Italy; 3 Gynecology and Obstetrics Unit, Department of Neuroscience, Reproductive Sciences and Dentistry, School of Medicine, University of Naples Federico II, Via Sergio Pansini, 5, 80131 NaplesNaples, Italy; 4 Gynecology and Obstetrics Unit, Department of Experimental and Clinical Medicine, Magna Graecia University of Catanzaro, Catanzaro, Italy; 5 Obstetrics and Gynecology Unit, Department of General Surgery and Medical Surgical Specialties, University of Catania, Catania, Italy; 6 Gynecology and Obstetrics Unit, Department of Medicine, Surgery and Dentistry “Schola Medica Salernitana”, University of Salerno, 84081 Baronissi, Italy

**Keywords:** bcl2, Immunohistochemistry, Leiomyoma, Prognosis, Smooth-muscle tumor

## Abstract

**Background:**

Uterine leiomyosarcoma (uLMS) may show loss of expression of B-cell lymphoma-2 (Bcl-2) protein. It has been suggested that Bcl-2 loss may both be a diagnostic marker and an unfavorable prognostic marker in uLMS.

**Objective:**

To define the diagnostic and prognostic value of Bcl-2 loss in uLMS through a systematic review and meta-analysis.

**Methods:**

Electronic databases were searched from their inception to May 2020 for all studies assessing the diagnostic and prognostic value of Bcl-2 loss of immunohistochemical expression in uLMS. Data were extracted to calculate odds ratio (OR) for the association of Bcl-2 with uLMS *vs* leiomyoma variants and smooth-muscle tumors of uncertain malignant potential (STUMP), and hazard ratio (HR) for overall survival; a *p* value < 0.05 was considered significant.

**Results:**

Eight studies with 388 patients were included. Loss of Bcl-2 expression in uLMS was not significantly associated with a diagnosis of uLMS *vs* leiomyoma variants and STUMP (OR = 2.981; *p* = 0.48). Bcl-2 loss was significantly associated with shorter overall survival in uLMS (HR = 3.722; *p* = 0.006). High statistical heterogeneity was observed in both analyses.

**Conclusion:**

Loss of Bcl-2 expression appears as a significant prognostic but not diagnostic marker in uLMS. The high heterogeneity observed highlights the need for further research and larger studies.

**Supplementary Information:**

The online version contains supplementary material available at 10.1007/s00404-022-06531-2.

## Introduction

Smooth-muscle tumors are the most common gynecologic neoplasms [1–4]. In most cases, they show that benign histological features, consisting of low mitotic index and absence of necrosis or cytologic atypia, and are labeled as leiomyoma [5, 6]. According to the Stanford criteria, a diagnosis of malignancy should be made in the presence of at least two of the following features: mitotic index > 10/10 high-power fields (HPF), at least moderate cytologic atypia, and coagulative tumor cell necrosis; in such cases, the tumor is labeled leiomyosarcoma [7]. Uterine leiomyosarcoma (uLMS) constitutes 60–70% of all uterine sarcomas [5]. The prognosis of uLMS is very poor, with less of half of patients being alive at 5 years [5]. Treatments for uLMS are non-specific and consist of chemotherapy and/or external beam radiotherapy [8].

The antiapoptotic protein B-cell lymphoma-2 (Bcl-2) has been proposed as diagnostic biomarker to distinguish uLMS from leiomyoma in challenging cases [9]. Indeed, Bcl-2 may show loss of immunohistochemical expression in uLMS, whereas it is almost always expressed in uterine leiomyomas [9]. In this regard, Bcl-2 loss might be a specific marker of malignancy, similarly to what was observed in endometrial pathology [10]. However, the reliability of Bcl-2 loss in differentiating uLMS from leiomyoma variants and smooth-muscle tumors of uncertain malignant potential (STUMP) is less clear. More interestingly, Bcl-2 has been proposed as a possible prognostic marker in uLMS [11–15]. In fact, it has been suggested that Bcl-2 loss identifies a subset of uLMS with more aggressive behavior [9, 11–15].

On this account, the aim of this study was to define whether Bcl-2 loss of expression is a significant diagnostic and prognostic marker in uLMS, by performing a systematic review and meta-analysis.

## Materials and methods

### Study protocol

This meta-analysis was designed based on previous studies [16, 17]. Each review stage (electronic search, study selection, data extraction, risk of bias within studies assessment, and data analysis) was independently performed by two authors; disagreements, if any, were solved by consensus among all authors. This review was reported following the PRISMA guidelines [18].

### Search strategy and study selection

Four electronic databases (Web of Sciences, Scopus, MEDLINE, and Google Scholar) were searched from their inception to May 2020 for all studies assessing the prognostic value of Bcl-2 immunohistochemical expression in uLMS. The following combination of text words was used: (uterine OR uterus OR gynecologic) AND (leiomyosarcoma) AND (immunohistochemistry OR immunohistochemical OR bcl2 OR bcl-2). Reference lists of relevant studies were also searched.

Exclusion criteria, defined a priori, were: sample size < 10, overlapping patient data, reviews.

### Data extraction

Data were extracted from primary studies by following the PICO [18]: “P” (population) consisted of patients with uLMS (for prognostic analysis) or with uLMS, leiomyoma variants or STUMP (for diagnostic analysis); “I” (intervention or risk factor) was the loss of Bcl-2 immunohistochemical expression; “C” (comparator) was a retained expression of Bcl-2; “O” (outcome) was the overall survival (for prognostic analysis) or a diagnosis of uLMS (for diagnostic analysis). Further extracted data were: country, period of enrollment, sample size, histological criteria for uLMS diagnosis, and methods for performing and interpreting immunohistochemistry, prognostic data.

### Risk of bias within studies assessment

The risk of bias within studies was assessed according to the QUADAS-2 [19]. Four crucial domains were assessed: (1) Patient selection, i.e., if patient selection criteria and period of enrollment were reported; (2) Index test, i.e., if methods for performing and interpreting immunohistochemistry were clearly reported and unbiased; (3) Reference standard, i.e., if diagnostic criteria for uLMS were clearly reported and unbiased (for diagnostic analysis), or if data about prognosis were reported and unbiased (for prognostic analysis); (4) Flow, i.e., if all eligible patients were assessed for Bcl-2 expression and prognosis (the latter one for prognostic analysis). The risk of bias was categorized as “low”, “unclear” or “high” as previously described [20, 21].

### Data analysis

Odds ratio (OR) with 95% confidence interval (CI) for the association of Bcl-2 loss with uLMS *vs* leiomyoma variants and STUMP was calculated for each study. Hazard ratio (HR) with 95% CI for overall survival was extracted or calculated from primary data for each study, as previously described [16]. If the primary study reported HR for Bcl-2 retained expression, we calculated the HR for Bcl-2 loss of expression as 1/HR. Pooled OR and HR were calculated using the random effect model of DerSimonian–Laird. Results were reported on forest plots. Statistical heterogeneity among studies was calculated using inconsistency index (*I*^2^) as previously described [22, 23]. The risk of bias across studies (publication bias) was assessed using a funnel plot reporting logarithm of OR/HR values on the x-axis and standard error on the y-axis.

Data analysis was performed using Comprehensive Meta-Analysis (Biostat, 14 North Dean Street, Englewood, NJ 07631, USA).

## Results

### Study selection and characteristics

Eight studies with a total sample size of 388 patients with uLMS were included [11–15, 24–26]; 64 leiomyoma variants (41 cellular leiomyomas and 23 leiomyomas with bizarre nuclei) and 22 STUMPs were also included. The process of study selection is shown in Supplementary Fig. 1. Four out of 8 studies evaluated the prognostic value of Bcl-2 in uLMS [12–14], 2 studies assessed Bcl-2 as a diagnostic marker of uLMS [25, 26], and 2 studies performed both diagnostic and prognostic analysis [11, 24]. Characteristics of the included studies are shown in Table 1; characteristics of patients and uLMS are shown in Table 2.

**Table 1:** Characteristics of the included studies

Study	Country	Period of enrollment	Sample size		
			LMS	STUMP	LM-V
Zhai [11]	Japan	Unclear	21	8	23
Bodner [24]	Austria	1990–2000	21	14	0
Leiser [12]	USA	1991–2004	36	0	0
Rath-Wolfson [25]	Israel	Unclear	10	0	20
D’Angelo [15]	Spain, Canada	1978–2008	84	0	0
Lusby [13]	USA	1989–2011	157	0	0
Stanescu [26]	Romania	2009–2012	6	0	21
Banas [14]	Poland	2000–2015	53	0	0

*LMS* leiomyosarcoma, *STUMP* smooth-muscle tumors of uncertain malignant potential, *LM-V* leiomyoma variants

**Table 2:** Characteristics of patients with uterine leiomyosarcoma

Study	Patient age, mean/median (range)	Menopausal status	Nulliparous, n	Cycle phase	Tumor diameter, cm mean/median (range)	Tumor stage (*n*)	Mitotic index, n/10HPF mean (range)	Adjuvant treatment, n (type)	Follow-up time, mean/median (range)	Bcl-2 antibody, clone (manufacturer)	Criterion to define Bcl-2 loss
Zhai et al. [11]	52 (32–70)	Not reported	Not reported	Not reported	Not reported	I (12), II (3), III (3), IV (3)	60 (14–133)	Not reported	23 (1–101) months	Unclear (Dako)	< 5%
Bodner et al. [24]	51 (36–78)	Not reported	Not reported	Not reported	Not reported	I (13), II (3), III (4), IV (1)	Not reported	10 (8 RT, 2 CT)	47 (1–228) months	124 (Dako)	< 10%
Leiser et al. [12]	53 (23–73)	Not reported	Not reported	Not reported	Not reported	I,II (19), III/IV (15)	Not reported	Not reported	47 (17–84) months	124 (Dako)	Weak in ≤ 80% or moderate/strong in ≤ 10%
Rath-Wolfson et al. [25]	61 (50–84)	Not reported	Not reported	Not reported	9 (4–15)	Not reported	Not reported	Not reported	Not assessed	Unclear	0% (individual data reported)
D’Angelo et al. [15]	51 (29–67)	Not reported	Not reported	Not reported	11 (3–35)	I (71), III (3), IV (3)	Not reported (5–19/10HPF)	31 (13 RT, 18 CT)	36 (3–96) months	100/D5 124 (Master Diagnostica)	< 50%
Lusby et al. [13]	52 (19–83)	Not reported	27	Not reported	11 (1–60)	Not reported	21 (1–83)	72 (24 RT, 8 CT)	35 (18–200) months	100 (Biogenex)	absent-to-weak intensity
Stanescu et al. [26]	55 (not reported)	Not reported	Not reported	Not reported	Not reported	I	13 (not reported)	Not reported	Not assessed	124 (Dako)	< 5%
Banas et al. [14]	51 (44–70)	30 pre- 23 post-	5	First phase	5 (2–15)	I (27), II (5), III (12), IV (9)	≤ 50 (38 patients), > 50 (15 patients)	32 (5 RT, 22 CT, 5 RT + CT)	Unclear	Unclear (Leica Biosystems)	< 10%

*RT* radiotherapy, *CT* chemotherapy

### Risk-of-bias assessment

For the “Patient selection” domain, unclear risk of bias was assigned to two studies (period of enrollment not reported) [11, 25], while low risk was assigned to the other studies.

For the “Index test” domain, low risk of bias was assigned to all included studies.

For the “reference standard”, unclear risk of bias was assigned to one study (follow-up duration not reported) [14], while low risk was assigned to the other studies.

For the “flow and timing” domain, unclear risk of bias was assigned to one study (only a subset of patients were assessed for the prognostic value of Bcl-2 expression) [13], while low risk was assigned to the other studies. Risk-of-bias results are presented graphically in Supplementary Fig. 2.

### Meta-analysis

**Fig. 1:**
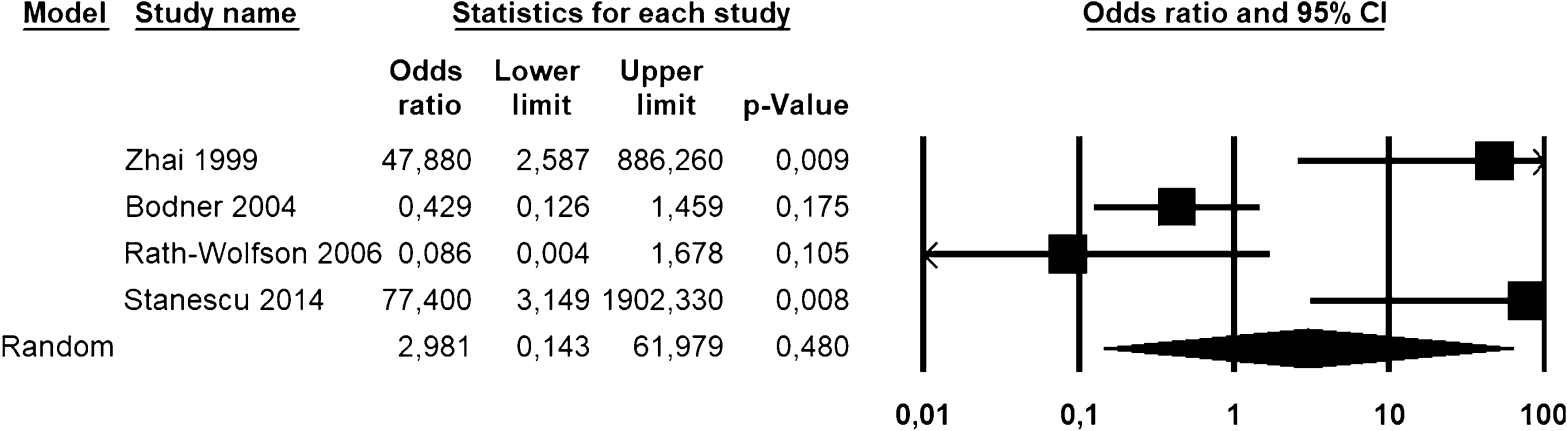
Forest plot reporting odds ratio (OR) values with 95% confidence interval (CI), for each study and as pooled estimate, for the association of Bcl-2 loss of immunohistochemical expression with a diagnosis of uterine leiomyosarcoma *vs* leiomyoma variants and STUMP

**Fig. 2:**
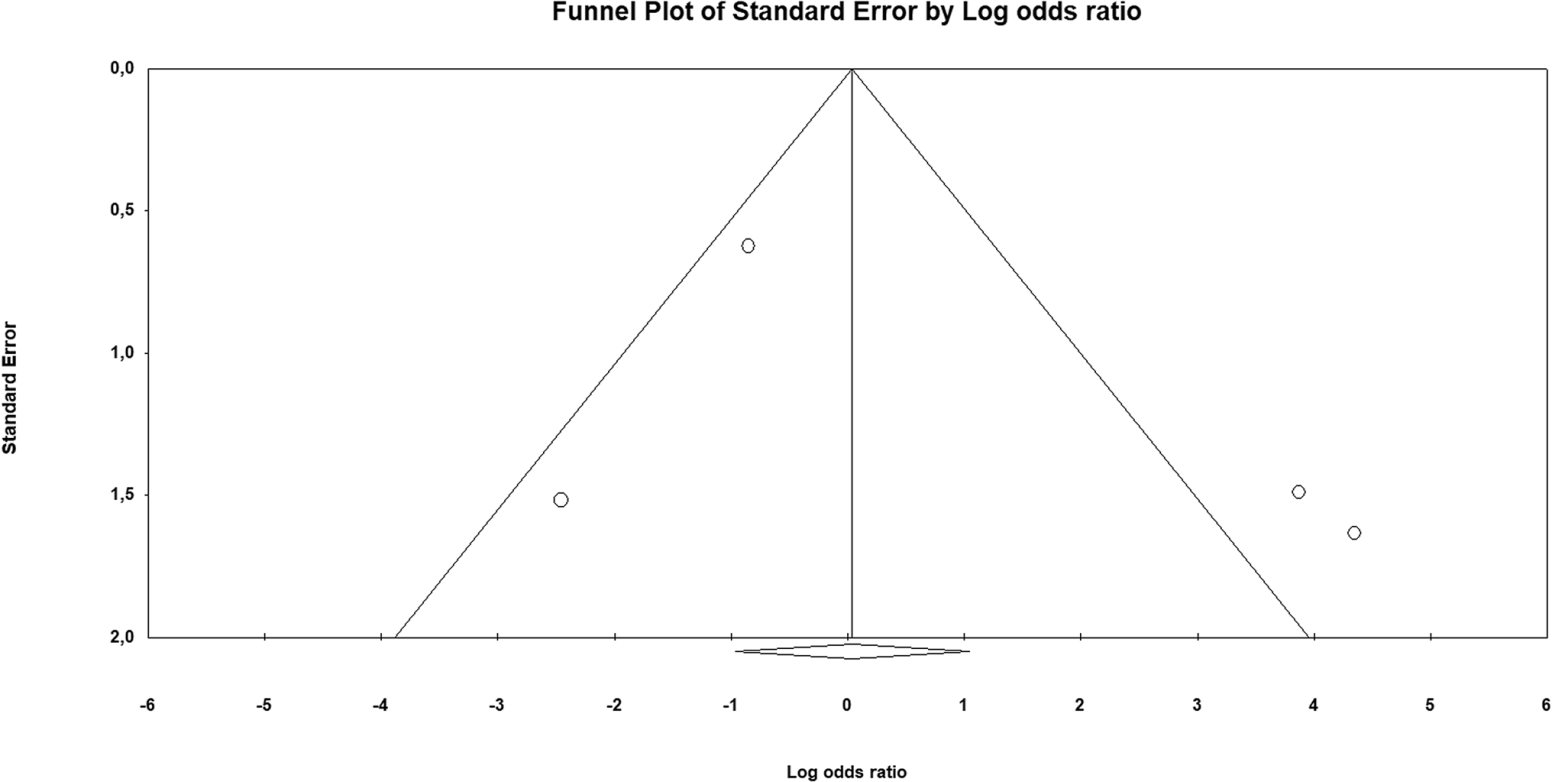
Funnel plot of logarithm of odds ratio by standard error for the assessment of the risk of bias across studies (publication bias). The asymmetrical distribution of the primary studies suggests the possibility of a publication bias

As a diagnostic marker, a loss of Bcl-2 immunohistochemical expression was not significantly associated with a diagnosis of uLMS *vs* leiomyoma variants and STUMP, with an OR of 2.981 (95% CI 0.143–61.979; p = 0.48) (Fig. 1). The statistical heterogeneity among studies was high (I^2^ = 83.598%). The funnel plot showed asymmetrical distribution of the primary studies, suggesting the possibility of a publication bias (Fig. 2).

**Fig. 3:**
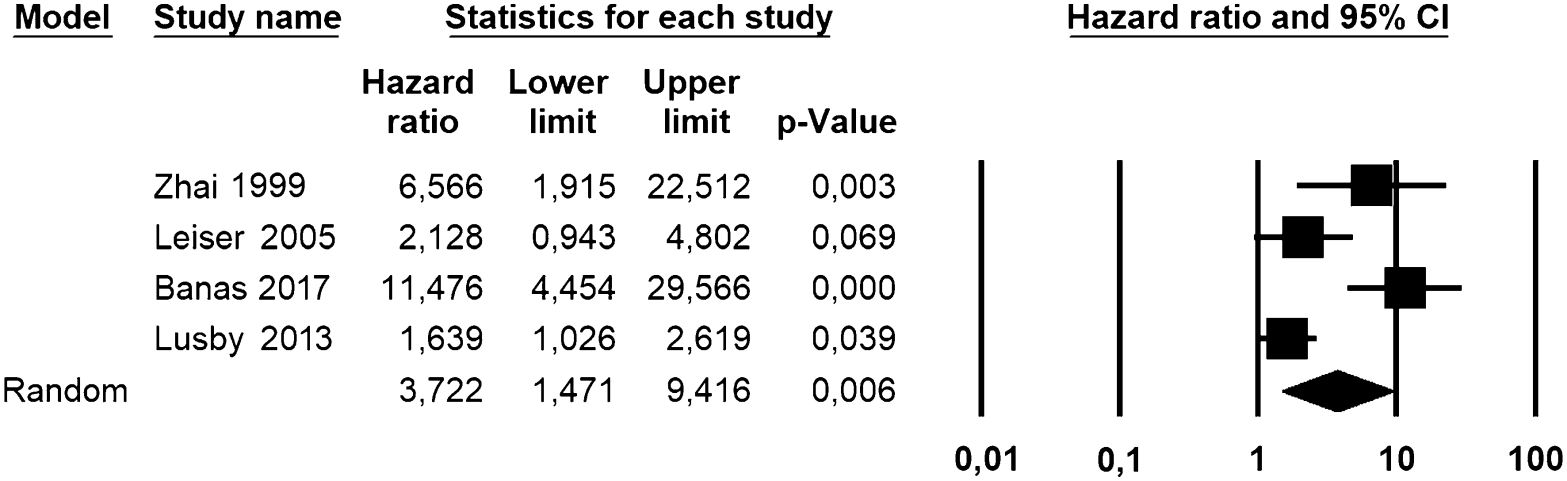
Forest plot reporting hazard ratio (HR) values with 95% confidence interval (CI), for each study and as pooled estimate, for the association of Bcl-2 loss of immunohistochemical expression with the risk of death in uterine leiomyosarcoma

**Fig. 4:**
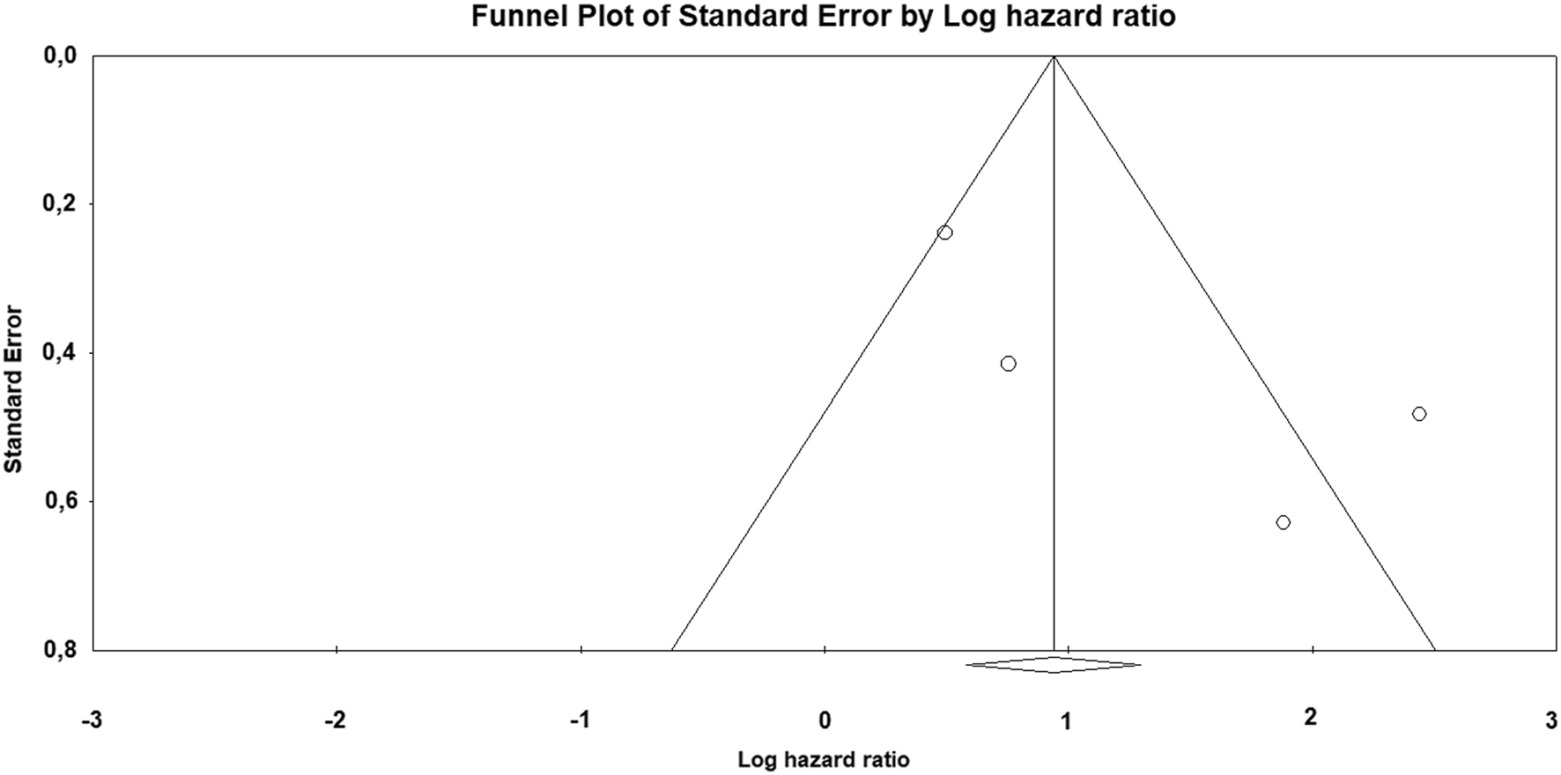
Funnel plot of logarithm of hazard ratio by standard error for the assessment of the risk of bias across studies (publication bias). The asymmetrical distribution of the primary studies suggests the possibility of a publication bias

As a prognostic marker, Bcl-2 loss was significantly associated with a decreased overall survival in uLMS, with an HR of 3.722 (95% CI 1.471–9.416; *p* = 0.006) (Fig. 3). The statistical heterogeneity among studies was high (*I*^2^ = 80.738%) and the funnel plot was asymmetrical (Fig. 4).

## Discussion

### Main findings and interpretation

This study showed that a loss of Bcl-2 immunohistochemical expression was significantly associated with shorter overall survival in uLMS, while it was not able to differentiate uLMS from leiomyoma variants and STUMP.

Bcl-2 protein is encoded by *BCL2* gene, a proto-oncogene that promotes tumorigenesis by inhibiting cell death; the antiapoptotic action of Bcl-2 protects cells against the effect of both endogenous and exogenous factors, including chemotherapeutic drugs and glucocorticoids [27]. On the other hand, the loss of Bcl-2 expression might indicate that the tumor shifted toward different pro-survival pathways associated with more aggressive behavior; on this account, Bcl-2 loss has been proposed as a possible marker of tumor aggressiveness [28].

With regard to uterine smooth-muscle tumors, the previous studies showed that the expression of Bcl-2 was higher in uterine leiomyomas than in the normal myometrium [29, 30]. Furthermore, Bcl-2 has been one of the main markers proposed to distinguish between benign and malignant uterine smooth-muscle tumors [9]. Zhai et al*.* found that Bcl-2 loss was significantly more common in uLMS than in uterine smooth-muscle tumors of uncertain malignant potential (STUMP) [11], while Bodner-Adler et al*.* found a significant difference between usual leiomyoma and both STUMP and uLMS, but not between STUMP and uLMS [24]. As seen in other human neoplasms, Bcl-2 has also been assessed as a prognostic marker in uLMS. In fact, several studies suggested that a loss of Bcl-2 expression in uLMS was associated with a worst prognosis [9, 11–15]. However, the small size of the individual studies prevents from draw conclusions about the diagnostic and prognostic value of Bcl-2 in uLMS.

Our study found that a loss of Bcl-2 immunohistochemical expression was not significantly associated with a diagnosis of uLMS. Remarkably, we compared uLMS to leiomyoma variants and STUMP, excluding usual leiomyomas. In fact, differentiating between usual leiomyoma and uLMS is not an issue, while leiomyoma variants and STUMP may raise the concern of malignancy [6, 7]. In fact, these lesions may show worrisome features (such as high mitotic index, increased cellularity, infiltrative borders, cytologic atypia, atypical mitoses, or necrosis) which makes it difficult to rule out a uLMS [5–7]; it is in these cases that a reliable diagnostic marker of uLMS should work. Our results do not mean that the expression of Bcl-2 is the same between uLMS, STUMP and leiomyoma variants. Instead, they suggest that a difference may exist (as indicated by the OR > 1), but is not statistically significant, making Bcl-2 inadequate as a diagnostic marker.

Regarding prognosis, we found that Bcl-2 loss was significantly associated with decreased overall survival in uLMS, with a 3.7-fold increase in the hazard of death. This result supports the usefulness of Bcl-2 as an immunohistochemical marker for the prognostic stratification in uLMS. In particular, the assessment of Bcl-2 might be useful in directing the choice of treatment in stage I (when adjuvant treatment is not mandatory) and in stages II-III, where the management of uLMS is not completely defined and includes the possibility of performing systemic therapy and/or external beam radiation therapy (EBRT) [8]. E.g., in patients at stage I, a loss of Bcl-2 expression might indicate the need for a treatment with systemic therapy or EBRT, while observation might be indicated for cases with retained Bcl-2 expression. In patients at stage II-III, Bcl-2 loss might require the combination of EBRT and systemic therapy. It is clear that conclusions cannot be drawn based on a small number of studies. Further studies are necessary to assess the potential role of Bcl-2 in the management of patients with uLMS.

Remarkably, Conconi et al*.* suggested that, in the STUMP category, Bcl-2 amplification could be associated with aggressive behavior, in contrast with its favorable significance in uLMS [31]. In this regard, it is necessary to correlate Bcl-2 with the whole histomorphologic pattern, to avoid misinterpretation of the immunohistochemical data. Furthermore, D’Angelo et al*.* found that the combined assessment of tumor size, mitotic index, Bcl-2, and ki67 lead to a still more precise prognostic stratification of uLMS [15]. On the account of these findings, further studies are encouraged to assess Bcl-2 on larger uLMS series, correlating its prognostic value with clinic-pathological data and with further immunohistochemical markers.

## Strengths and limitations

To our knowledge, this is the first meta-analysis assessing the diagnostic and prognostic value of Bcl-2 in uLMS. A limitation to our results may be the low number of included studies and patients. Two studies could not be included in the prognostic analysis, because they did not report extractable data [15, 24]; however, since both showed a significant prognostic value for Bcl-2, they would not affect the significance of the results if they could be included in the analysis.

Another limitation may be the high statistical heterogeneity found. Causes for such heterogeneity might lie in different criteria adopted to interpret Bcl-2 immunohistochemistry, with particular regard to the definition of loss of expression. In fact, several different thresholds of intensity and distribution of immunostaining were used. Furthermore, the anti-Bcl-2 antibody was not the same in all studies. Possible confounding factors regarding patients’ characteristics, uLMS features, adjuvant treatment, and immunohistochemical methods are reported in Table 2. Unfortunately, some relevant data, such as menopausal status and menstrual cycle phase, were not provided by most studies.

Finally, a limitation of our meta-analysis may be the possibility of a publication bias, as suggested by the asymmetry of the funnel plots. Therefore, even though the prognostic significance of Bcl-2 was consistent among the published studies, it cannot be excluded that the studies with negative findings were not submitted/published. We hope that our results will encourage further studies to assess this point.

## Conclusion

Loss of Bcl-2 immunohistochemical expression does not appear able to differentiate uLMS from leiomyoma variants and STUMP, resulting therefore not useful as a diagnostic marker. By contrast, Bcl-2 loss appears as a significant unfavorable prognostic marker in uLMS. Given the wide availability and low costs of immunohistochemistry, the assessment Bcl-2 expression might easily be introduced in the common practice for the prognostic stratification of uLMS, and might be useful in directing the patient management. However, limitations such as the low number of included studies, the high statistical heterogeneity, and the possibility of a publication bias prevent from drawing conclusions. Further studies are encouraged in this regard.

## Supplementary Information

Below is the link to the electronic supplementary material.

Supplementary file 1 Figure 1. Flow diagram of studies identified in the systematic review (Prisma template [Preferred Reporting Item for Systematic Reviews and Meta-analyses]). PNG 18 KB)

Supplementary file 2 Figure 2. Assessment of risk of bias. Summary of risk of bias for each study; Plus sign: low risk of bias; minus sign: high risk of bias; question mark: unclear risk of bias. (PNG 11 KB)
