# Supplementary figures and images for: Diagnostic and prognostic value of Bcl-2 in uterine leiomyosarcoma
Source: Arch Gynecol Obstet. 2022 Mar 28;307(2):379–86. doi: 10.1007/s00404-022-06531-2 (PMC9918573; doi:10.1007/s00404-022-06531-2)

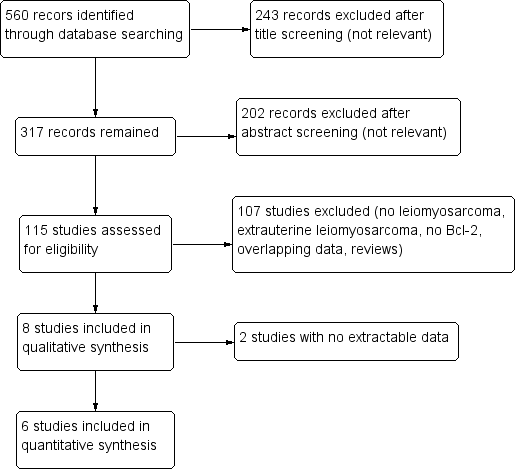

Supplement: Supplementary file 1 — Figure 1. Flow diagram of studies identified in the systematic review (Prisma template [Preferred Reporting Item for Systematic Reviews and Meta-analyses]). PNG 18 KB) [file 404_2022_6531_MOESM1_ESM.png]

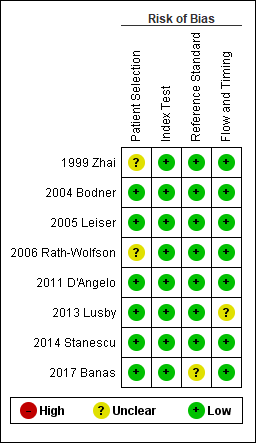

Supplement: Supplementary file 2 — Figure 2. Assessment of risk of bias. Summary of risk of bias for each study; Plus sign: low risk of bias; minus sign: high risk of bias; question mark: unclear risk of bias. (PNG 11 KB) [file 404_2022_6531_MOESM2_ESM.png]
